# Obstructive azoospermia secondary to bilateral epididymal cystadenomas in a patient with von Hippel-Lindau

**DOI:** 10.1016/j.eucr.2019.100922

**Published:** 2019-05-29

**Authors:** Patrick T. Gomella, Paul Shin, Ramaprasad Srinivasan, W. Marston Linehan, Mark W. Ball

**Affiliations:** a Urologic Oncology Branch, National Cancer Institute, Bethesda, MD, USA; b Shady Grove Fertility, Rockville, MD, USA

**Keywords:** VHL, von-Hippel Lindau, Epididymal obstruction, Obstructive azoospermia, Sperm retrieval

## Abstract

von Hippel-Lindau (VHL) is a heritable cancer syndrome associated with findings in multiple organ systems. Male patients can be affected by epididymal cystadenomas which are benign tumors localized to the epididymis. While benign, these tumors can cause pain and in very rare circumstances can have an effect on fertility especially when present bilaterally. We present a case of a young man with obstructive azoospermia secondary to bilateral cystadenomas, with a focus on his work-up and management.

## Introduction

von Hippel-Lindau (VHL) is a heritable cancer syndrome associated with findings in multiple organ systems. Individuals are at risk for developing benign and malignant lesions of the kidneys, adrenals, pancreas, central nervous system, eyes and reproductive organs. Epididymal cystadenomas (EC) are benign lesions that occur in 25–60% of affected men.^1^ Given their striking microscopic similarity to metastatic clear cell renal cell carcinoma, if excision is pursued, diagnosis relies on clinical history and immunohistochemical staining (typically CK7-positive and CD10-negative) for accurate histologic interpretation. Typically, epididymal cystadenomas are slow growing painless epidydimal nodules, however some patients report pain with these lesion (approximately 10%) and desire excision. Given the usual benign clinical course of these lesions, they are followed conservatively, unless the patient is symptomatic. While rare, cystadenomas, especially when bilateral, can be a cause of male factor infertility, with only several reported cases in the literature. Details of the previous case reports in the literature are summarized in Table 1. Herein we present an additional case of a patient with azoospermia in the setting of bilateral epididymal cystadenomas with a focus on his work-up and management.

**Table 1:** Previous Reported Cases of Infertility in setting of Bilateral Epididymal Cystadenomas.

Authors	Journal	Publication Year	Patient Age	Clinical Details	Management
Crisp et al.	British Journal of Urology	1975	32	Primary Infertility	Scrotal Exploration; Vasogram; Epididymal and Testicular Biopsy
Tsuda et al.	Cancer	1976	30 & 39	Infertility & Scrotal Swelling	*Not discussed*
deSouza Andrade et al.	Journal of Urology	1985	29	Attempted to concieve for 14 months	Lesion excision followed by Testicular Biopsy
Witten et al.	Journal of Urology	1985	24	Primary Infertility	Testicular Exploration; no sperm seen on microsurgical dissection of epididymis; Testicular biopsy performed with sperm retrieved
Pozza et al.	Urologia Internationalis	1994	28	Primary Infertility	Scrotal Exploration

## Case details

A 30-year-old male with VHL presented for enrollment in a clinical trial at our institution. Prior to initiation of the trial, he pursued sperm banking to preserve his fertility if the trial effected spermatogenesis. A properly collected semen specimen was analyzed and despite normal volume (2.7 cc), he was found to be azoospermic. An azoospermia work-up was initiated which included a focused physical exam, blood work (testosterone, FSH, LH, karyotype, y chromosome microdeletion analysis) and a scrotal ultrasound. All patients at our institution are enrolled in active research protocols which include acknowledgement of informed consent for the responsible use of the data obtained in the course of their care, additionally no identifiable information is included in this case report.

**Fig. 1:**
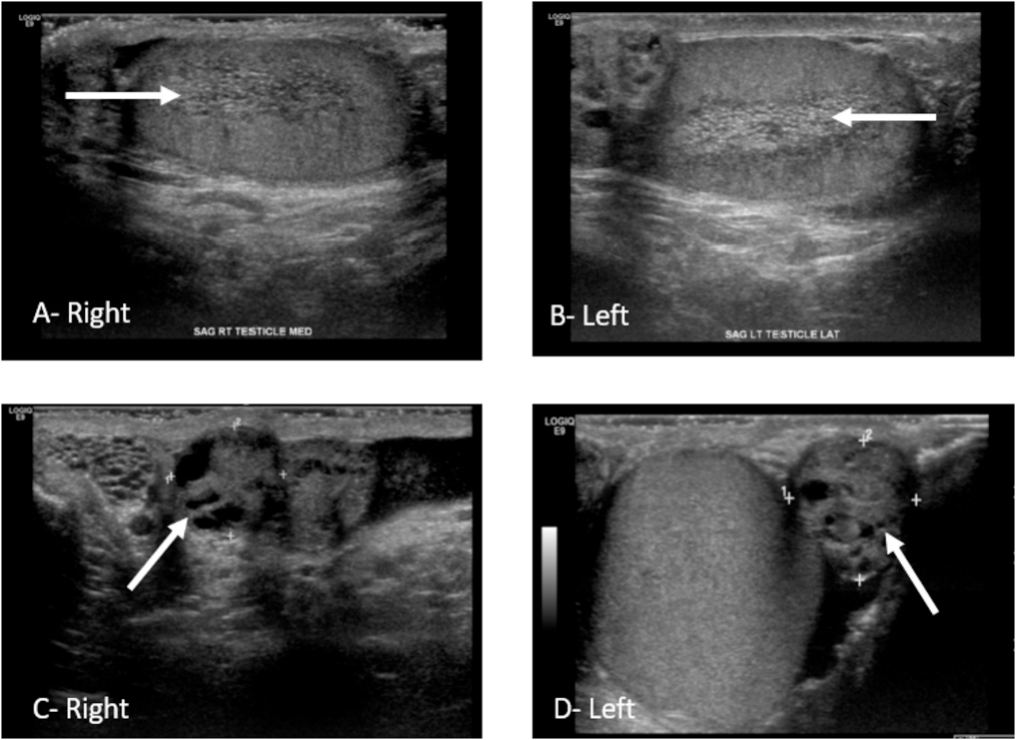
Ultrasound Images. Right and Left Testicles noted dilated rete testes (Arrows-Images A, B); Bilateral epididymal lesions with mostly solid components and small cystic areas consistent with cystadenomas (Arrows-Images C, D).

Details of the laboratory evaluation are included in Table 2 and were all within normal limits. On physical exam, he had bilateral painless firm lesions involving the heads of both epididymides consistent with epididymal cystadenomas and testicles with normal texture. Scrotal ultrasound confirmed bilateral lobulated cystic lesion in both epididymides with bilateral dilated rete testes with high normal testicular volumes (23 cc right; 19 cc left). Representative ultrasound images are included in Fig. 1. He was diagnosed with obstructive azoospermia and counseled on his options. He elected sperm retrieval and cryopreservation to allow him to pursue intracytoplasmic sperm injection or in vitro fertilization in the future. Under general anesthesia, he first underwent epididymal aspiration which noted ample fluid with crystals but no sperm. A testicular biopsy was performed with ample sperm recovered which were able to be preserved.

**Table 2:** Laboratory evaluation.

Lab Test	Result
FSH	4.9 U/L
LH	3.6 U/L
SHBG	33 nmol/L (13–71)
Total Testosterone	433 ng/dL (262–1593)
Albumin	4.6 g/dL (3.5–5.2)
Free Testosterone	8.6 ng/dL (7.4–22.6)
Karyotype	Normal
Y Chromosome microdeletion	No deletion identified

## Discussion

It is unclear what percentage of male VHL patients are afflicted with male factor infertility. The focus on family planning in patients with a confirmed pathogenic mutation of the VHL gene focuses mostly on pre-natal testing and pre-implantation genetic diagnosis, which have been used successfully in patients with hereditary cancer syndromes.^2^ When comparing VHL patients to unaffected siblings, reproductive ability was only shown to be slightly reduced (8.1 children/100 reproductive years vs 9.79/100 reproductive years) in those effected with VHL.^3^ In male VHL patients who are affected with infertility, a high index of suspicion is necessary to evaluate for anatomic obstruction in patients with epididymal cystadenomas.

Scrotal ultrasound can be used to follow epididymal cystadenomas in affected individuals and was an important component in the evaluation of azoospermia in this patient. On ultrasound, ECs typically show as an ovoid mass in the head of the epididymis which is mostly solid with some cystic areas. In one series, 23% of VHL patients were found to have dilated rete testes but only in patients age 30 and older. No specific fertility work up was performed in this series; however, several patients with bilateral EC were able to father children naturally.^4^ While dilated rete testes are associated with distal obstruction, it does not confirm obstruction and would need to be further investigated with a semen analysis if azoospermia or fertility issues are encountered in a patient with such findings.

In a patient with obstructive azoospermia and bilateral epididymal cystadenomas who desires children, the optimal treatment strategy has not been established. Symptomatic ECs are most often treated with epididymectomy, which itself renders the treated side azoospermic. While cyst excision can be performed, as in with spermatocelectomy, epididymal injury can occur and cyst excision is not guaranteed to relieve the obstruction. Standard approaches to obstructive azoospermia which include sperm retrieval via percutaneous or open biopsy of the epididymis and/or testicle or bypass/repair of the obstructed area using microsurgical techniques are the preferred management strategies. While reconstruction may be a viable option in a patient desiring to try and conceive naturally, sperm retrieval for use with assisted reproductive techniques has a near 100% success in this clinical scenario for collection of viable sperm.^5^
